# Complete genome sequence of chicharrita del maíz iflavirus 1, a virus previously identified from transcriptomic data of the corn leafhopper (*Dalbulus maidis*)

**DOI:** 10.1128/mra.00788-25

**Published:** 2026-02-27

**Authors:** Juliana Osse de Souza, Alejandro Olmedo-Velarde

**Affiliations:** 1 Department of Plant Pathology, Entomology and Microbiology, Iowa State University 1177, Ames, Iowa, USA; DOE Joint Genome Institute, Berkeley, California, USA

**Keywords:** iflavirus, *Dalbulus maidis*, corn leafhopper, insect viruses

## Abstract

We report a complete genome of chicharrita del maiz iflavirus 1 from the United States, determined by RNAseq, PCR, and RACE in RNA extracted from *Dalbulus maidis* (corn leafhopper). The genome encodes a 3,306-amino-acid polyprotein with conserved capsid and replication domains, expanding knowledge of iflavirus diversity in hemipteran vectors.

## ANNOUNCEMENT

**Fig 1:**
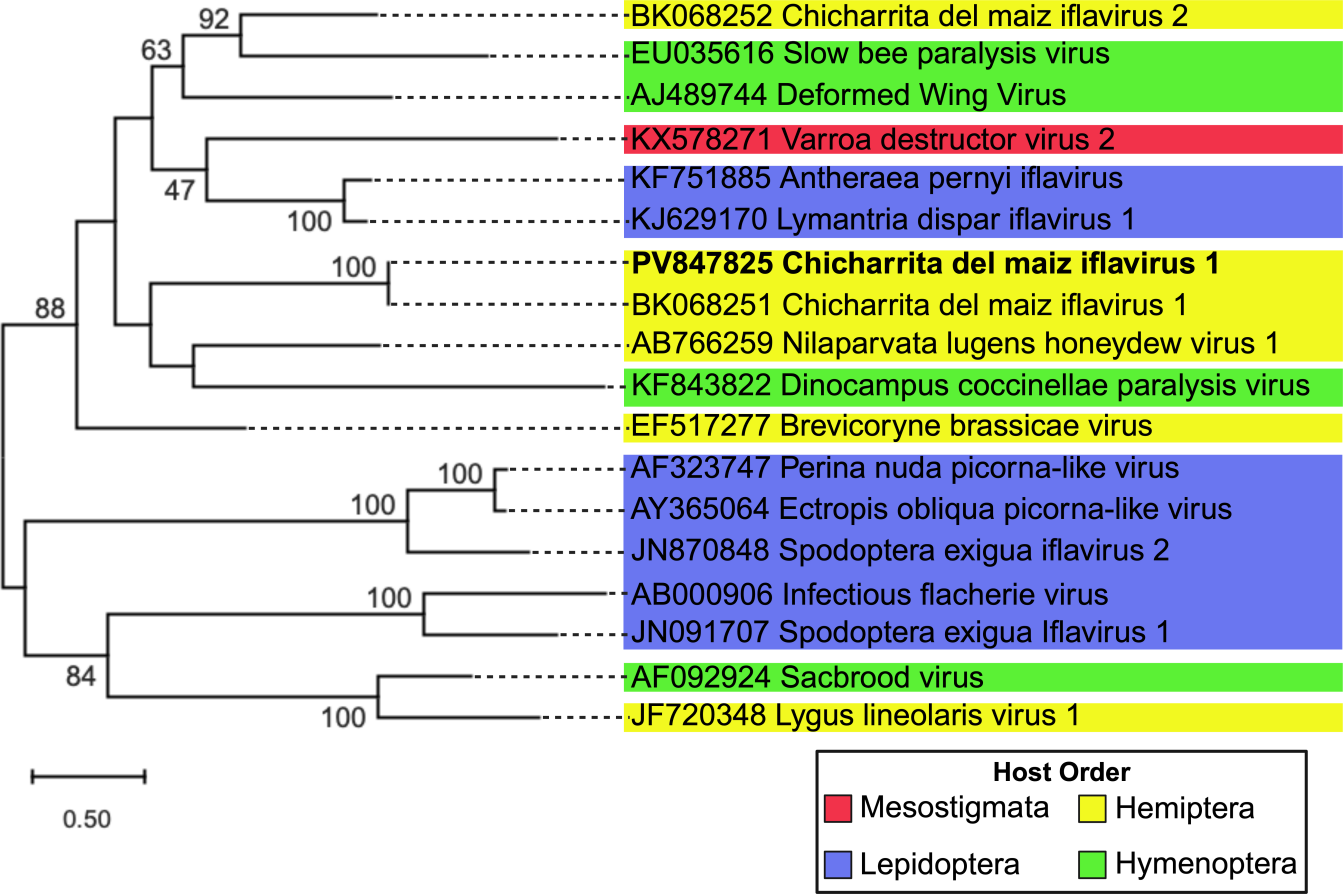
Phylogenetic relationships of the amino acid sequence of the capsid protein (CP) of chicharrita del maiz iflavirus 1 (ChMIfV1) with their homologs from related virus members in the family *Iflaviridae* infecting other arthropods. Pairwise sequence alignment was performed using MUSCLE (8), the best-fit amino acid substitution model was inferred using ModelTest NG (9), and the phylogenetic tree was constructed using the maximum likelihood (amino acid substitution model *LG+I + G4+F*) method implemented in MEGA12 (10).

The family *Iflaviridae* contains small, non-enveloped viruses that infect arthropods and cause outcomes ranging from asymptomatic to developmental and lethal effects (1). Adult *Dalbulus maidis* were collected from two commercial corn fields in Story City, Iowa, United States, in September 2024 using sweep nets. Insects were stored in 70% ethanol at −20°C until morphological identification (2). Total RNA was extracted from one to three whole insects using the Synergy 2.0 Plant DNA Extraction kit (OPS Diagnostics), as per the kit instructions, but omitting the RNase digestion step. RNA from multiple samples was pooled to generate a composite sample used for complementary DNA (cDNA) library preparation with the TruSeq Stranded Total RNA with Ribo-Zero H/M/R Gold kit (Illumina). Paired-end sequencing (150 bp) was performed on an Illumina NovaSeqX platform, generating ~70 million reads. Bioinformatic analyses were performed as previously detailed (3). Briefly, quality-filtered reads were *de novo* assembled using rnaviralSPAdes v.4.2.0 (4), and virus contigs were annotated using the NCBI online BLASTx against the Virus protein database using default parameters. A contig of 11,098 nucleotides (110x coverage) was identified and showed >95% amino acid identity to chicharrita del maíz iflavirus 1 (ChMIfV1, BK068251), a virus *in silico* identified in public data sets of *D. maidis* originating from Argentina, the United States, and Brazil (5). RT-PCR using ChMIfV1-specific RNA-dependent RNA polymerase (RdRp) primers (Table 1) confirmed its presence. To obtain the complete genome, we used overlapping amplicons (Table 1), combined with 5′ and 3′ rapid amplification of cDNA ends (RACE). RACE was performed using dsRNA polyadenylated with *Escherichia coli* poly (A) polymerase and terminal deoxynucleotidyl transferase (New England Biolabs) (6). Amplicons were sequenced using Sanger and Nanopore sequencing technologies (Table 1). Nanopore sequencing was performed by PlasmidSaurus using the primer-free v14 library prep chemistry on a MinION Mk1D device (Oxford Nanopore Technologies) and basecalled with Guppy (SUP model). Geneious Prime 2025.1.3 software was used for the reference-based assembly using accession no. BK068251, manual curation, and annotation of the genome, including the prediction of protein cleavage sites. Protein conserved domains were annotated using the NCBI Conserved Domain Search Tool. The full genome of ChMIfV1 (11,146 nt, PV847825) is polyadenylated and contains 5′ and 3′ untranslated regions (UTRs) of 975 and 250 nt, respectively, flanking a single open reading frame encoding a 3,306-amino-acid (aa) polyprotein. Conserved domains included three capsid proteins (aa 324–512, 655–722, and 997–1,148), an RNA helicase (aa 1,550–1,656), and an RdRp domain (aa 2,952–3,266). Comparisons using the NCBI online BLASTp tool showed the highest similarity to ChMIfV1 (DBA56841, query coverage 100%; identity 98.79%) assembled from public transcriptomes, followed by Nephotettix cincticeps positive-stranded RNA virus-1 (UFP63464, query coverage 96%; identity 63.44%). A maximum likelihood phylogenetic analysis of capsid proteins of several *Iflaviridae* members (Fig. 1) placed the Iowa isolate in a clade with the previously reported ChMIfV1 isolate (5) and Nilaparvata lugens honeydew virus 1 found infecting the brown planthopper (7). This complete genome provides a foundation for future studies on virus diversity and host interactions in *D. maidis*.

**TABLE 1:** Primers used for genome validation and rapid amplification of complementary ends (RACE) of chicharrita del maiz iflavirus 1 (ChMIfV1)

Oligo Name	Sequence (5′ to 3′)	Product size (bp)	Sequencing method^*a*^
ChMIfV1_11,218F_3RACE	TGAGGACCAGGATTATGCAGCT		ONT
ChMIfV1_464R_5RACE	ACGTTTATTTCAATTCGCCACAAAACC		ONT
ChMIfV1_P1_1,924R	TAGCCCACTCCTAAGCCACC	1,492	ONT
ChMIfV1_P1_432F	AGCTTTCATTGTTGGAGATGGTGT		
ChMIfV1_P2_1,770F	GGATTGCCGCCCATAAGG	1,217	ONT
ChMIfV1_P2_2,987R	CCGATGTTGTTGGCTTATCTCT		
ChMIfV1_P3_2,843F	TTTATTTCATTTGAAAATACCCAGT	1,192	ONT
ChMIfV1_P3_4,035R	AATACATAAACATAATCACCATCA		
ChMIfV1_P4_3,908F	GGTACAATGTATTATTTGAAATGTAA	1,489	ONT
ChMIfV1_P4_5,397R	TTAGTGCATACGGATATAACCCC		
ChMIfV1_P5_5,258F	TTAAATGCTTTATCTCATTGTT	1,009	ONT
ChMIfV1_P5_6,267R	TTTCTATCACATTCTGCCAT		
ChMIfV1_P6_6,107F	GGAACTAAGTATTGGGAAAATT	1,437	
ChMIfV1_P6_7,544R	TAATTCCTACACAAACTTCAATT		Sanger
ChMIfV1_P7_7,431F	TAGAGGCTATTAGAAATATTAGAGA	1,527	ONT
ChMIfV1_P7_8,958R	AACAACCGGCGAAGGATATC		
ChMIfV1_P8_10,371R	ATCATATTACTACAACTAAATCGGT	1,576	ONT
ChMIfV1_P8_8,795F	ACTATTTTTGCTACCAAACCAGA		
ChMIfV1_P9_10,232F	TCCAAAGAAAAAGTAGCAGT	1,112	Sanger
ChMIfV1_P9_11,344R	TCACCTGAAACTCCACAG		
ChMIfV1_RdRp_F	GAGTAGGCGCTGGTATACCG	477	Sanger
ChMIfV1_RdRp_R	CCCAGCCCTGCATATGAAGG		

^*a*^ Each PCR fragment was sequenced using Oxford Nanopore Technology (ONT) through Plasmidsaurus or by Sanger sequencing at the Iowa State University DNA Facility.

## Data Availability

The RNAseq data set for *D. maidis* from Iowa has been deposited in NCBI SRA under accession no. PRJNA1282137. The whole-genome sequence for chicharrita del maiz iflavirus 1 (ChMIfV1) from Iowa was deposited under accession number PV847825.
